# Supplementary material for: Comparable in vivo joint kinematics between self-reported stable and unstable knees after TKA can be explained by muscular adaptation strategies: A retrospective observational study
Source: eLife. 2023 Apr 26;12:e85136. doi: 10.7554/eLife.85136 (PMC10205082; doi:10.7554/eLife.85136)
Supplement: Supplementary file 1. [file elife-85136-supp1.docx]

|  | **Level walking** | | **Downhill walking** | | **Stair descent** | |
| --- | --- | --- | --- | --- | --- | --- |
|  | Stable | Unstable | Stable | Unstable | Stable | Unstable |
| **Medial A-P [mm]** | -17.8 ± 2.1 | -15.5 ± 2.9 | -17.9 ± 1.6 | -15.4 ± 2.9 | -18.2 ± 1.9 | -16.8 ± 1.4 |
| **Lateral A-P [mm]** | -17.6 ± 1.9 | -15.5 ± 2.7 | -17.2 ± 1.2 | -15.3 ± 2.9 | -17.9 ± 1.6 | -17.2 ± 1.1 |
| **Flex/ex [°]** | -2.0 ± 2.5 | 1.5 ± 8.6 | -2.6 ± 3.5 | 3.0 ± 8.2 | 5.3 ± 2.7 | 9.0 ± 6.7 |
| **Ab/add [°]** | 0.1 ± 0.3 | 0.2 ± 0.5 | 0.3 ± 0.4 | 0.2 ± 0.4 | 0.0 ± 0.7 | 0.3 ± 0.5 |
| **Int/ext [°]** | 0.4 ± 1.2 | 0.7 ± 2.2 | 1.2 ± 1.5 | 1.2 ± 1.9 | 1.3 ± 1.8 | 0.8 ± 2.3 |

*Supplementary file 1: Mean ± standard deviation of the anterior-posterior (A-P) tibiofemoral positions for the medial and lateral condyles, as well as differences between flexion/extension (Flex/ex), adduction/abduction (Ab/add), and internal/external (Int/ext) rotation angles are reported for the stable and unstable groups at the instant of heel-strike of level walking, downhill walking, and stair descent.*
