# Supplementary material for: Comparable in vivo joint kinematics between self-reported stable and unstable knees after TKA can be explained by muscular adaptation strategies: A retrospective observational study
Source: eLife. 2023 Apr 26;12:e85136. doi: 10.7554/eLife.85136 (PMC10205082; doi:10.7554/eLife.85136)
Supplement: Supplementary file 2. — Bold values indicate the comparison of the same muscle between stable and unstable groups. RF: rectus femoris, VM: vastus medial, VL: vastus lateral, TA: tibialis anterior, HM: hamstrings medial, HL: hamstring lateral, GM: gastrocnemius medial, GL: gastrocnemius lateral. [file elife-85136-supp2.docx]

| **ANOVA** | **GL_stable** | **GM_stable** | **HL_stable** | **HM_stable** | **RF_stable** | **TA_stable** | **VL_stable** | **VM_stable** |
| --- | --- | --- | --- | --- | --- | --- | --- | --- |
| **GL_unstable** | 0.658 | - | - | - | - | - | - | - |
| **GM_unstable** | - | 0.87007 | - | - | - | - | - | - |
| **HL_unstable** | - | - | 0.67617 | - | - | - | - | - |
| **HM_unstable** | - | - | - | 0.3568 | - | - | - | - |
| **RF_unstable** | - | - | - | - | 0.94069 | - | - | - |
| **TA_unstable** | - | - | - | - | - | 0.36318 | - | - |
| **VL_unstable** | - | - | - | - | - | - | 0.94945 | - |
| **VM_unstable** | - | - | - | - | - | - | - | 0.56587 |

*Supplementary file 2: Post-hoc pair-wise comparisons of one-way ANOVA results on hamstrings-dominant classified synergy module during stair descent. Bold values indicate the comparison of the same muscle between stable and unstable groups. RF: rectus femoris, VM: vastus medial, VL: vastus lateral, TA: tibialis anterior, HM: hamstrings medial, HL: hamstring lateral, GM: gastrocnemius medial, GL: gastrocnemius lateral.*
